# Supplementary material for: Noise Expands the Response Range of the Bacillus subtilis Competence Circuit
Source: PLoS Comput Biol. 2016 Mar 22;12(3):e1004793. doi: 10.1371/journal.pcbi.1004793 (PMC4803322; doi:10.1371/journal.pcbi.1004793)
Supplement: S1 Text — (PDF) [file pcbi.1004793.s001.pdf]

# S1 Text for "Noise expands the response range of the *Bacillus subtilis* competence circuit"

Andrew Mugler[1,◕], Mark Kittisopikul[2,3,◕], Luke Hayden[1,4], Jintao Liu[3], Chris H. Wiggins[5], Gürol M. Süel[3,‡,†], Aleksandra M. Walczak[6,‡,*],

1 Department of Physics and Astronomy, Purdue University, West Lafayette, IN 47907, USA
2 Department of Biophysics, University of Texas Southwestern Medical Center, Dallas, TX 75390, USA
3 Division of Biological Sciences, University of California San Diego, CA 92093, USA
4 Division of Natural Sciences, Indiana Wesleyan University, Marion, IN 46953, USA
5 Department of Applied Physics and Applied Mathematics, Columbia University, New York, NY 10027, USA
6 Laboratoire de Physique Théorique, CNRS, Université Pierre et Marie Curie and École Normale Supérieure, 75005 Paris, France

◕ These authors contributed equally to this work.
‡ Joint corresponding authors.
† gsuel@ucsd.edu
* awalczak@lpt.ens.fr

## 1 Parameter values

The parameter values used in the model in the main text are taken from [13,15,16], where they have been optimized to agree with a number of experimental observations, including the existence of competence events, the duration of these events, and the robustness of events to a wide range of stress levels. They are first given below in dimensionless form, which is sufficient to establish their deterministic dynamical properties. To account for intrinsic noise and to compare with experiments, several physical quantities are then specified to establish molecule numbers and timescales. The conversion to the remaining model parameters, and their resulting values, are then given below.

Native circuit:

| Dimensionless parameters | Physical quantities | Remaining parameters |
|---|---|---|
| $a_s = 0$ | Molecule numbers: | $\alpha_k$ (varied) |
| $b_k = 0.3$ | $\Gamma_k = 100$ | $\alpha_s = a_s\Gamma_s\delta = 0/\text{s}$ |
| $b_s = 3$ | $\Gamma_s = 1$ | $\beta_k = b_k\Gamma_k\delta_k = 0.03/\text{s}$ |
| $k_0 = 0.2$ | | $\beta_s = b_s\Gamma_s\delta_s = 0.003/\text{s}$ |
| $k_1 = 1/30$ | Timescales: | $k_k = k_0\Gamma_k = 20$ |
| $\Delta_k = 0.1$ | $\delta_k = 0.001/\text{s}$ | $k_s = k_1\Gamma_k = 3.3$ |
| $\Delta_s = 0.1$ | $\delta_s = 0.001/\text{s}$ | $\lambda_k = \Delta_k\delta_k = 10^{-4}/\text{s}$ |
| $h = 2$ | | $\lambda_s = \Delta_s\delta_s = 10^{-4}/\text{s}$ |
| $p = 5$ | | |

SynEx circuit:

| Dimensionless parameters | Physical quantities | Remaining parameters |
|---|---|---|
| $a_m = 0.3$<br>$b_k = 15$<br>$b_m = 10$<br>$\mu = 1$<br>$h = 2$<br>$p = 2$ | Molecule numbers:<br>$k_k = 10$<br>$k_m = 5$<br><br>Timescales:<br>$\lambda_k = 10^{-4}$/s<br>$\lambda_m = 10^{-4}$/s | $\alpha_k$ (varied)<br>$\alpha_m = a_m k_m \lambda_m = 1.5 \times 10^{-4}$/s<br>$\beta_k = b_k k_k \lambda_k = 0.015$/s<br>$\beta_m = b_m k_m \lambda_m = 0.005$/s<br>$\delta = \mu \lambda_k / k_m = 2 \times 10^{-5}$/s |

Note that in [13,15,16] the higher values $\Gamma_k = 25000$ and $\Gamma_s = 20$ (native), and $k_k = 5000$ and $k_m = 2500$ (SynEx) are used to establish molecule number. We use the lower values here to elucidate the effects of intrinsic noise. This assumption is relaxed in the next section, where we return to the high-number regime. In the main text, we discuss how the effects of noise are robust to this choice.

## 2 Linear stability analysis of the deterministic model

Here, for the dynamics in Fig. 4 and S1, we list the fixed points and the eigenvalues of the Jacobian matrix evaluated at each fixed point:

| | Fig. 4A (left) | Fig. 4A (right) | Fig. S1C and D | Fig. 4B (left) | Fig. 4B (right) |
|---|---|---|---|---|---|
| Fixed Point 1 | $\lambda_1 = -0.045$<br>$\lambda_2 = -0.10$ | $\lambda_1 = -0.44$<br>$\lambda_2 = -0.81$ | $\lambda_1 = -0.03 + 0.52i$<br>$\lambda_2 = -0.03 - 0.52i$ | $\lambda_1 = -0.25$<br>$\lambda_2 = -0.88$ | $\lambda_1 = -0.6 + 1.9i$<br>$\lambda_2 = -0.6 - 1.9i$ |
| 2 | $\lambda_1 = 0.04$<br>$\lambda_2 = -0.10$ | $\lambda_1 = 0.43$<br>$\lambda_2 = -0.69$ | | | |
| 3 | $\lambda_1 = 0.05 + 0.28i$<br>$\lambda_2 = 0.05 - 0.28i$ | $\lambda_1 = 2.1$<br>$\lambda_2 = 0.54$ | | | |
| Regime | excitable | excitable | monostable (damped oscillations) | monostable | monostable (damped oscillations) |

We see that for both circuits at low stress (columns 1 and 2), there is one stable fixed point (negative eigenvalues) and two unstable fixed points (where at least one eigenvalue has positive real part), consistent with excitable behavior. For both circuits at high stress (columns 3 and 5), there is one fixed point, and its eigenvalues are complex with negative real part, indicating damped oscillations. For the native circuit at very high stress (column 4), there is one fixed point, and its eigenvalues are real and negative, indicating a monostable state.

## 3 Relaxing the model assumptions

To relax the first two simplifying assumptions made in the main text, we consider a stochastic model that has high molecular copy numbers, and that accounts explicitly for the mRNA dynamics and the dynamics of competitive degradation. The model follows from our earlier work [16], and consists of a set of coupled chemical reactions for each circuit. Due to the complexity of the model, we do not solve the master equation explicitly, but rather we simulate the dynamics using the Gillespie algorithm [50].

For the native circuit, the reactions and rates are:

| Reaction | Rate | Additional parameters |
|---|---|---|
| $P^{const}_{ComK} \to P^{const}_{ComK} + mRNA_{ComK}$ | $k_1$ (varied) | $k_2 = 0.19/s$ |
| $P_{ComK} \to P_{ComK} + mRNA_{ComK}$ | $k_2 n^h/(k_k^h + n^h)$ | $k_5 = 0.0015/s$ |
| $mRNA_{ComK} \to mRNA_{ComK} + ComK$ | $k_3 = 0.2/s$ | $k_{11} = 2 \times 10^{-6}/s$ |
| $P^{const}_{ComS} \to P^{const}_{ComS} + mRNA_{ComS}$ | $k_4 = 0/s$ | $k_{13} = 4.5 \times 10^{-6}/s$ |
| $P_{ComS} \to P_{ComS} + mRNA_{ComS}$ | $k_5/[1 + (n/k_s)^p]$ | $k_k = 5000$ |
| $mRNA_{ComS} \to mRNA_{ComS} + ComS$ | $k_6 = 0.2/s$ | $k_s = 833$ |
| $mRNA_{ComK} \to \emptyset$ | $k_7 = 0.005/s$ | $h = 2$ |
| $ComK \to \emptyset$ | $k_8 = 10^{-4}/s$ | $p = 5$ |
| $mRNA_{ComS} \to \emptyset$ | $k_9 = 0.005/s$ | $\Omega = 1.66\ \mu m^3$ |
| $ComS \to \emptyset$ | $k_{10} = 10^{-4}/s$ | $M_T = 500$ |
| $MecA + ComK \to MecA_K$ | $k_{11}/\Omega$ | |
| $MecA_K \to MecA + ComK$ | $k_{-11} = 5 \times 10^{-4}/s$ | |
| $MecA_K \to MecA$ | $k_{12} = 0.05/s$ | |
| $MecA + ComS \to MecA_S$ | $k_{13}/\Omega$ | |
| $MecA_S \to MecA + ComS$ | $k_{-13} = 5 \times 10^{-5}/s$ | |
| $MecA_S \to MecA$ | $k_{14} = 4 \times 10^{-5}/s$ | |

Here $P_{gene}$ denotes the promoter of the corresponding gene (constitutive or regulated), and $MecA_K$ and $MecA_S$ represent the complex of MecA bound to ComK and ComS, respectively. As in the main text, $n$ is the number of ComK molecules per cell. $\Omega$ represents the cell volume, and $M_T$ gives the total number of MecA molecules. Upon adiabatically eliminating the faster mRNA dynamics and the MecA dynamics, one obtains the model in the previous section, except with the larger molecule numbers $\Gamma_k = 25000$ and $\Gamma_s = 20$ [13]. The control parameter here, $k_1$, is related to the control parameter in the reduced model, $\alpha_k$, via $k_1 = k_7\alpha_k/k_3$ [13].

For the SynEx circuit, the reactions and rates are:

| Reaction | Rate | Additional parameters |
|---|---|---|
| $P^{const}_{ComK} \to P^{const}_{ComK} + mRNA_{ComK}$ | $k_1$ (varied) | $k_2 = 0.19/s$ |
| $P_{ComK} \to P_{ComK} + mRNA_{ComK}$ | $k_2 n^h/(k_k^h + n^h)$ | $k_4 = 0.0625/s$ |
| $P^{const}_{MecA} \to P^{const}_{MecA} + mRNA_{MecA}$ | $k_3 = 0.0019/s$ | $k_7 = 4 \times 10^{-8}/s$ |
| $P_{MecA} \to P_{MecA} + mRNA_{MecA}$ | $k_4 n^p/(k_m^p + n^p)$ | $k_k = 5000$ |
| $mRNA_{ComK} \to mRNA_{ComK} + ComK$ | $k_5 = 0.2/s$ | $k_m = 2500$ |
| $mRNA_{MecA} \to mRNA_{MecA} + MecA$ | $k_6 = 0.2/s$ | $h = 2$ |
| $MecA + ComK \to MecA$ | $k_7/\Omega$ | $p = 2$ |
| $mRNA_{ComK} \to \emptyset$ | $k_8 = 0.005/s$ | $\Omega = 1.66\ \mu m^3$ |
| $mRNA_{MecA} \to \emptyset$ | $k_9 = 0.005/s$ | |
| $MecA \to \emptyset$ | $k_{10} = 10^{-4}/s$ | |
| $ComK \to \emptyset$ | $k_{11} = 10^{-4}/s$ | |

Once again, $P_{gene}$ denotes the promoter of the corresponding gene, $n$ is the number of ComK molecules per cell, and $\Omega$ represents the cell volume. Upon adiabatically eliminating the faster mRNA dynamics, one obtains the model in the previous section, except with the larger molecule numbers $k_k = 5000$ and $k_m = 2500$ [16]. The control parameter here, $k_1$, is related to the control parameter in the reduced model, $\alpha_k$, via $k_1 = k_8\alpha_k/k_5$.

To relax the third simplifying assumption made in the main text, we consider a stochastic model of the SynExSlow circuit. The model follows from our earlier work [16], and is similar to the stochastic model in the main text, except that there are three species with dynamic molecule numbers: ComK ($n$), MecA ($m$),

and ComS ($\ell$). The production rate functions are, respectively,

$$g_n = \alpha_k + \frac{\beta_k n^h}{k_k^h + n^h}, \qquad q_n = \alpha_m + \frac{\beta_m n^p}{k_m^p + n^p}, \qquad y_n = \alpha_s + \frac{\beta_s n^h}{k_s^h + n^h}, \tag{1}$$

and the degradation rate functions are, respectively,

$$r_{nm\ell} = \frac{\delta_k m}{1 + n/\Gamma_k + \ell/\Gamma_s} + \lambda_k, \qquad s = \lambda_m, \qquad u_{nm\ell} = \frac{\delta_s m}{1 + n/\Gamma_k + \ell/\Gamma_s} + \lambda_s. \tag{2}$$

The parameter values are [16]:

| Parameters | |
|---|---|
| $\alpha_k$ (varied) | $\beta_s = 0.5$/s |
| $\alpha_m = 0.075$/s | $\Gamma_k = 25000$ |
| $\alpha_s = 0.5$/s | $\Gamma_s = 20$ |
| $\delta_k = \delta_s = 2 \times 10^{-6}$/s | $k_k = 5000$ |
| $\lambda_k = \lambda_m = \lambda_s = 10^{-4}$/s | $k_m = 2500$ |
| $\beta_k = 7.5$/s | $k_s = 500$ |
| $\beta_m = 2.5$/s | $h = p = 2$ |

Note that these values correspond to the high-molecule-number regime of the native and SynEx models of the main text, and thus this model of the SynExSlow circuit also relaxes the first simplifying assumption of low molecule number.

Finally, we note that at these parameter values, the deterministic analog of this model predicts that as a function of the control parameter $\alpha_k$, the excitable regime transitions directly into a damped oscillatory (mono-stable) regime, where two of the three eigenvalues of the Jacobian matrix are complex, and all have negative real part. That is, there is no standard oscillatory regime. Therefore, we define a heuristic boundary $\alpha_k^{(2)} = 0.15$/s after which the damping is clearly evident within the first 24 hours of the deterministic dynamics (see Fig. S7). The fact that the stochastic dynamics exhibit sustained oscillations in the absence of a deterministically oscillatory regime, even beyond this heuristic boundary (Fig. S7), indicates that the ability of noise to induce oscillations in the SynExSlow circuit is especially strong.

# 4 Spectral solution to the master equation

We write the master equation (Eqn. 1 in the main text) as

$$\frac{dp_{nm}}{dt} = -\left(\hat{\mathcal{L}}_n[g_n, r_{nm}] + \hat{\mathcal{L}}_m[q_n, s_{nm}]\right) p_{nm}. \tag{3}$$

Here $-\hat{\mathcal{L}}$ is the linear birth-death operator, whose action on the probability distribution $p$ is described by

$$-\hat{\mathcal{L}}_n[g_n, r_n]p_n = g_{n-1}p_{n-1} + r_{n+1}(n+1)p_{n+1} - (g_n + r_n n)p_n, \tag{4}$$

where in general on an operator (here, $\hat{\mathcal{L}}$) we use a subscript to denote the sector ($n$ or $m$) on which it acts. Explicitly, then, the master equation reads

$$\begin{aligned} \frac{dp_{nm}}{dt} &= g_{n-1}p_{n-1,m} + r_{n+1,m}(n+1)p_{n+1,m} - (g_n + r_{nm}n)p_{nm} \\ &\quad + q_n p_{n,m-1} + s_{n,m+1}(m+1)p_{n,m+1} - (q_n + s_{nm}m)p_{nm}. \end{aligned} \tag{5}$$

We will now derive the spectral decomposition of the master equation by introducing the generating function. For intuition, we will first introduce the generating function in the context of the one-dimensional system described by Eqn. 4, then extend our results to the full master equation.

The generating function is an expansion in a complete set of states, indexed by molecule number, for which the probabilities provide the expansion coefficients. Denoting the states abstractly as $|n\rangle$, the generating function is defined

$$|G\rangle \equiv \sum_n p_n |n\rangle, \tag{6}$$

where the sum runs from 0 to $\infty$ (as do all sums hereafter unless otherwise specified). Summing Eqn. 4 against $|n\rangle$ yields

$$\begin{aligned} -\hat{\mathcal{L}}|G\rangle &= \sum_n g_{n-1}p_{n-1}|n\rangle + \sum_n r_{n+1}(n+1)p_{n+1}|n\rangle - \sum_n g_n p_n|n\rangle - \sum_n r_n n p_n|n\rangle & (7)\\ &= \sum_n g_n p_n|n+1\rangle + \sum_n r_n n p_n|n-1\rangle - \sum_n g_n p_n|n\rangle - \sum_n r_n n p_n|n\rangle, & (8)\end{aligned}$$

where the second step shifts the sum without penalty (i) in the first term, since $p_{-1} = 0$, and (ii) in the second term, since it vanishes for $n = 0$. Eqn. 8 benefits from the introduction of two additional sets of operators: (i) operators corresponding to the evaluation of the production and degradation functions at particular values of $n$,

$$\begin{aligned} \hat{g}|n\rangle &\equiv g_n|n\rangle, & (9)\\ \hat{r}|n\rangle &\equiv r_n|n\rangle, & (10)\end{aligned}$$

and (ii) raising and lowering operators that correspond to the shifts in $n$ caused by birth and death,

$$\begin{aligned} \hat{a}^+|n\rangle &\equiv |n+1\rangle, \qquad & \langle n|\hat{a}^+ &= \langle n-1|, & (11)\\ \hat{a}^-|n\rangle &\equiv n|n-1\rangle, \qquad & \langle n|\hat{a}^- &= n\langle n+1|. & (12)\end{aligned}$$

In Eqns. 11-12, for completeness, we have also presented the operators' actions to the left, which derive from the orthonormality condition $\langle n|n'\rangle = \delta_{nn'}$. In terms of the above operators, Eqn. 8 becomes

$$\begin{aligned} -\hat{\mathcal{L}}|G\rangle &= \sum_n p_n\hat{a}^+\hat{g}|n\rangle + \sum_n p_n\hat{a}^-\hat{r}|n\rangle - \sum_n p_n\hat{g}|n\rangle - \sum_n p_n\hat{a}^+\hat{a}^-\hat{r}|n\rangle, & (13)\\ &= \left(\hat{a}^+\hat{g} + \hat{a}^-\hat{r} - \hat{g} - \hat{a}^+\hat{a}^-\hat{r}\right)\sum_n p_n|n\rangle, & (14)\\ &= -\left(\hat{a}^+ - 1\right)\left(\hat{a}^-\hat{r} - \hat{g}\right)|G\rangle. & (15)\end{aligned}$$

Eqn. 15 reveals the form of the birth-death operator in generating function space.

We now generalize Eqn. 15 to the two-dimensional master equation of the model. Defining the two-dimensional generating function,

$$|G\rangle \equiv \sum_{nm} p_{nm}|n,m\rangle, \tag{16}$$

the master equation becomes

$$|\dot{G}\rangle = -\left[\left(\hat{a}_n^+ - 1\right)\left(\hat{a}_n^-\hat{r}_{nm} - \hat{g}_n\right) + \left(\hat{a}_m^+ - 1\right)\left(\hat{a}_m^-\hat{s}_{nm} - \hat{q}_n\right)\right]|G\rangle, \tag{17}$$

where dot denotes the time derivative, and as before the subscripts on operators denote the sectors on which they act.

The key insight of the spectral method is that a master equation such as Eqn. 17 can be simplified significantly by expansion in a wisely chosen set of eigenfunctions. Since Eqn. 17 describes two coupled birth-death

processes, we choose to expand in the eigenfunctions of two *uncoupled* birth-death processes – that is, processes with *constant* production rates $\bar{g}$ and $\bar{q}$ and degradation rates $\bar{r}$ and $\bar{s}$, respectively. Again for intuition we begin in one dimension, for which the operator describing a constant-rate birth-death process is given by Eqn. 15:

$$-\hat{\mathcal{L}}|G\rangle = -\left(\hat{a}^+ - 1\right)\left(\hat{a}^-\bar{r} - \bar{g}\right)|G\rangle = -\bar{r}\hat{b}^+\hat{b}^-|G\rangle. \tag{18}$$

In the second step we have defined the shifted raising and lowering operators

$$\hat{b}^+ \equiv \hat{a}^+ - 1, \tag{19}$$
$$\hat{b}^- \equiv \hat{a}^- - \bar{g}/\bar{r}. \tag{20}$$

The operator $\hat{b}^+\hat{b}^-$ is a number operator whose eigenvalues are integers, which we call $j$; thus the eigenvalue relation for the constant-rate birth-death operator is

$$-\hat{\mathcal{L}}|j\rangle = -\bar{r}j|j\rangle. \tag{21}$$

Accordingly, $\hat{b}^+$ and $\hat{b}^-$ raise and lower the eigenstates as $\hat{a}^+$ and $\hat{a}^-$ do the $|n\rangle$ states (Eqn. 11-12):

$$\hat{b}^+|j\rangle = |j+1\rangle, \qquad \langle j|\hat{b}^+ = \langle j-1|, \tag{22}$$
$$\hat{b}^-|j\rangle = j|j-1\rangle, \qquad \langle j|\hat{b}^- = j\langle j+1|. \tag{23}$$

As we will see, the spectral method exploits the expansion of the generating function in these eigenstates $|j\rangle$.

Returning to two dimensions, it is clear that we would like to write the coupled master equation in terms of the uncoupled rates, which we do by adding them to and subtracting them from Eqn. 17:

$$\begin{aligned}
|\dot{G}\rangle &= -\left[\left(\hat{a}_n^+ - 1\right)\left(\hat{a}_n^-[\hat{r}_{nm} + \bar{r} - \bar{r}] - \hat{g}_n - \bar{g} + \bar{g}\right) + \left(\hat{a}_m^+ - 1\right)\left(\hat{a}_m^-[\hat{s}_{nm} + \bar{s} - \bar{s}] - \hat{q}_n - \bar{q} + \bar{q}\right)\right]|G\rangle & (24)\\
&= -\left[\left(\hat{a}_n^+ - 1\right)\left(\hat{a}_n^-\bar{r} - \bar{g} - \hat{a}_n^-\hat{\gamma}_{nm} + \hat{\Gamma}_n\right) + \left(\hat{a}_m^+ - 1\right)\left(\hat{a}_m^-\bar{s} - \bar{q} - \hat{a}_m^-\hat{\lambda}_{nm} + \hat{\Lambda}_n\right)\right]|G\rangle & (25)\\
&= -\left[\bar{r}\hat{b}_n^+\hat{b}_n^- - \hat{b}_n^+\left(\hat{b}_n^- + \frac{\bar{g}}{\bar{r}}\right)\hat{\gamma}_{nm} + \hat{b}_n^+\hat{\Gamma}_n + \bar{s}\hat{b}_m^+\hat{b}_m^- - \hat{b}_m^+\left(\hat{b}_m^- + \frac{\bar{q}}{\bar{s}}\right)\hat{\lambda}_{nm} + \hat{b}_m^+\hat{\Lambda}_n\right]|G\rangle. & (26)
\end{aligned}$$

In the second step we define operators which capture the deviations between the constants and the coupled rates,

$$\hat{\Gamma}_n \equiv \bar{g} - \hat{g}_n, \qquad \hat{\Lambda}_n \equiv \bar{q} - \hat{q}_n, \tag{27}$$
$$\hat{\gamma}_{nm} \equiv \bar{r} - \hat{r}_{nm}, \qquad \hat{\lambda}_{nm} \equiv \bar{s} - \hat{s}_{nm}, \tag{28}$$

and in the third step we write the raising and lowering operators in terms of their shifted counterparts:

$$\hat{b}_n^+ \equiv \hat{a}_n^+ - 1, \qquad \hat{b}_m^+ \equiv \hat{a}_m^+ - 1, \tag{29}$$
$$\hat{b}_n^- \equiv \hat{a}_n^- - \bar{g}/\bar{r}, \qquad \hat{b}_m^- \equiv \hat{a}_m^- - \bar{q}/\bar{s}. \tag{30}$$

We now expand the generating function in the eigenstates of $\hat{b}_n^+\hat{b}_n^-$ and $\hat{b}_m^+\hat{b}_m^-$, with eigenvalues $j$ and $k$, respectively,

$$|G\rangle = \sum_{jk} G_{jk}|j,k\rangle, \tag{31}$$

which makes Eqn. 26

$$\sum_{jk}\dot{G}_{jk}|j,k\rangle = -\sum_{jk}G_{jk}\left[\bar{r}j - \hat{b}_n^+\hat{b}_n^-\hat{\gamma}_{nm} - \frac{\bar{g}}{\bar{r}}\hat{b}_n^+\hat{\gamma}_{nm} + \hat{b}_n^+\hat{\Gamma}_n + \bar{s}k - \hat{b}_m^+\hat{b}_m^-\hat{\lambda}_{nm} - \frac{\bar{q}}{\bar{s}}\hat{b}_m^+\hat{\lambda}_{nm} + \hat{b}_m^+\hat{\Lambda}_n\right]|j,k\rangle. \tag{32}$$

Projecting from the left with the state $\langle j,k|$,

$$\begin{aligned}\dot{G}_{jk} &= -(\bar{r}j+\bar{s}k)G_{jk} - \sum_{j'} G_{j'k}\langle j|\hat{b}_n^{+}\hat{\Gamma}_n|j'\rangle - \sum_{j'k'} G_{j'k'}\langle j,k|\hat{b}_m^{+}\hat{\Lambda}_n|j',k'\rangle \\ &\quad + \sum_{j'k'} G_{j'k'}\langle j,k|\hat{b}_n^{+}\hat{b}_n^{-}\hat{\gamma}_{nm}|j',k'\rangle + \frac{\bar{g}}{\bar{r}}\sum_{j'k'} G_{j'k'}\langle j,k|\hat{b}_n^{+}\hat{\gamma}_{nm}|j',k'\rangle \\ &\quad + \sum_{j'k'} G_{j'k'}\langle j,k|\hat{b}_m^{+}\hat{b}_m^{-}\hat{\lambda}_{nm}|j',k'\rangle + \frac{\bar{q}}{\bar{s}}\sum_{j'k'} G_{j'k'}\langle j,k|\hat{b}_m^{+}\hat{\lambda}_{nm}|j',k'\rangle, \end{aligned} \tag{33}$$

and noting the actions of $\hat{b}^{+}$ and $\hat{b}^{-}$ to the left (Eqns. 29-30) yields the dynamics of the expansion coefficients,

$$\dot{G}_{jk} = -(\bar{r}j+\bar{s}k)G_{jk} - \sum_{j'}\left[\Gamma_{j-1,j'}G_{j'k} + \Lambda_{jj'}G_{j',k-1}\right] + \sum_{j'k'}\left[j\gamma_{jj'}^{kk'} + \frac{\bar{g}}{\bar{r}}\gamma_{j-1,j'}^{kk'} + k\lambda_{jj'}^{kk'} + \frac{\bar{q}}{\bar{s}}\lambda_{jj'}^{k-1.k'}\right]G_{j'k'}, \tag{34}$$

where the deviations, rotated into eigenspace, have become matrices and tensors:

$$\Gamma_{jj'} \equiv \langle j|\hat{\Gamma}_n|j'\rangle = \sum_n \langle j|n\rangle(\bar{g}-g_n)\langle n|j'\rangle, \qquad \Lambda_{jj'} \equiv \langle j|\hat{\Lambda}_n|j'\rangle = \sum_n \langle j|n\rangle(\bar{q}-q_n)\langle n|j'\rangle, \tag{35}$$

$$\begin{aligned}\gamma_{jj'}^{kk'} &\equiv \langle j,k|\hat{\gamma}_{nm}|j',k'\rangle & \lambda_{jj'}^{kk'} &\equiv \langle j,k|\hat{\lambda}_{nm}|j',k'\rangle \\ &= \sum_{nm}\langle j|n\rangle\langle k|m\rangle(\bar{r}-r_{nm})\langle n|j'\rangle\langle m|k'\rangle, & &= \sum_{nm}\langle j|n\rangle\langle k|m\rangle(\bar{s}-s_{nm})\langle n|j'\rangle\langle m|k'\rangle. \end{aligned} \tag{36}$$

With Eqn. 34, we have turned the original master equation (Eqn. 17) into a linear algebraic equation, involving only matrix and tensor multiplication. In steady state Eqn. 34 can be solved perturbatively by treating the first term on the right-hand side as larger than the others and iterating to convergence.

Algorithmically, then, the procedure prescribed by the spectral method is the following. First we compute the deviation matrices and tensors from the given rate functions $g_n$, $r_{nm}$, $q_n$, and $s_{nm}$, and our chosen 'gauges' $\bar{g}$, $\bar{r}$, $\bar{q}$, and $\bar{s}$. Then we solve for the steady state of Eqn. 34 by iteration to obtain the expansion coefficients $G_{jk}$. Finally, we compute the probability distribution by taking the inverse transform:

$$p_{nm} = \sum_{jk} G_{jk}\langle n|j\rangle\langle m|k\rangle. \tag{37}$$

The probability distribution provides the complete stochastic description of the steady-state process. Note that both the deviation matrices/tensors and the probability distribution are computed via multiplication against the eigenmodes $\langle n|j\rangle$ and $\langle m|k\rangle$ or their conjugates $\langle j|n\rangle$ and $\langle k|m\rangle$; these are efficiently precomputed via recursive update rules.

For the SynEx circuit, the dynamics of the spectral expansion coefficients are simplified because we have $r_{nm}\to r_m$ and $s_{nm}\to\bar{s}$:

$$\dot{G}_{jk} = -(\bar{r}j+\bar{s}k)G_{jk} - \sum_{j'}\left[\Gamma_{j-1,j'}G_{j'k} + \Lambda_{jj'}G_{j',k-1}\right] + \sum_{k'}\left[j\gamma_{kk'}G_{jk'} + \frac{\bar{g}}{\bar{r}}\gamma_{kk'}G_{j-1,k'}\right], \tag{38}$$

where

$$\Gamma_{jj'} \equiv \sum_n \langle j|n\rangle(\bar{g}-g_n)\langle n|j'\rangle, \qquad \Lambda_{jj'} \equiv \sum_n \langle j|n\rangle(\bar{q}-q_n)\langle n|j'\rangle, \tag{39}$$

$$\gamma_{kk'} \equiv \sum_m \langle k|m\rangle(\bar{r}-r_m)\langle m|k'\rangle. \tag{40}$$

We see that the sparser coupling of the degradation terms (compared to the native circuit) has left us with a linear algebraic equation involving only matrices, not tensors. The solution follows the steps outlined above for the native circuit.

The spectral method is more efficient than other solution techniques by many orders of magnitude [48]. However, we find that here it becomes numerically unstable at sufficiently high numbers. Therefore, at high copy numbers, we solve for the steady state of the original master equation (Eqn. 3) by iteration. This is less efficient but more numerically stable. Specifically, we tile the columns of $p_{nm}$ into one vector $P_i$, and write the master equation as

$$\frac{dP_i}{dt} = \sum_{i'} M_{ii'} P_{i'} = \sum_{i'} \left(-D_i \delta_{ii'} + N_{ii'}\right) P_{i'}, \tag{41}$$

where $M_{ii'}$ is the reshaped operator in Eqn. 3, and $-D_i$ and $N_{ii'}$ are its diagonal and non-diagonal components, respectively. In steady state, we have

$$P_i = D_i^{-1} \sum_{i'} N_{ii'} P_{i'}, \tag{42}$$

which we solve iteratively until convergence.

# 5 Chromosomal alterations and antibiotic resistance

In this study, strains of the *Bacillus subtilis* strain PY79 were genetically altered by homologous combination, resulting in a single copy of each promoter-gene pair integrated into the bacterial chromosome.

The **Native strain** is a variant from a previous study [13]. This strain includes an IPTG inducible promoter, $P_{hyperspank}$ that drives $ComK$ production together with the native *comK* gene, which is left intact. This was introduced into the *amyE* gene locus and was selected for using spectinomycin resistance, $Sp^R$. Furthermore, a construct containing $P_{comG} - cfp$ and $P_{comS} - yfp$ was inserted into the sacA gene locus using chloramphenicol, $Cm^R$, as an antibiotic selection marker. A table that summarizes the chromosomal alterations, synthetic gene insertions, and antibiotic resistance in the order of transformations is below.

| Locus::Construct (Antibiotic Resistance) |
|---|
| $amyE :: P_{hyperspank} - comK(Sp^R)$ |
| $sacA :: P_{comG} - cfp, P_{comS} - yfp(Cm^R)$ |

The **SynExSlow** strain is similar to the one described in [16] with a few changes. For this study, we wanted to be able to tune $ComK$ production using IPTG as in the native strain above. However, the SynExSlow strain as previously described used the *hyperspank* promoter to regulate the basal level of *ComS*. Here we replaced the original *comS* regulation, $amyE :: P_{hyperspank} - comS$, with *comS* expression from a ribosomal promoter, $P_{rpsD} - comS$ with constitutive expression similar to the expression previously described. This was inserted into the *gltA* gene locus together with $P_{comG} - comS$, which slows down the synthetic competence differentiation, using phleomycin ($Plm^R$) as a selection marker. Originally, the $P_{comG} - comS$ promoter-gene construct was inserted into the *sacA* gene locus, but in this variant only the $pSac - cm - P_{comG-Kbox1} - mecA^{xp}$ is inserted into the *sacA* locus with chloramphenicol resistance ($Cm^R$) as a selection marker due to better transformation efficiency. The final difference is the identical $P_{hyperspank} - comK$ construct inserted into the *amyE* gene locus with spectinomycin resistance ($Sp^R$) as was used with the Native strain. The knock-out of the $srfA$ gene locus containing *comS* was done by inserting with $comS :: neo, P_{comG} - cfp$ with neomycin and kanamycin resistance ($Neo^R/Kan^R$) as in the original SynExSlow.

This is summarized according to the actual order of transformations in the following table:

| Locus::Construct (Antibiotic Resistance) |
| --- |
| $amyE :: P_{hyperspank} - comK(Sp^R)$ |
| $sacA :: P_{comG-Kbox1} - mecA^{xp}(Cm^R)$ |
| $\Delta srfA, comS :: P_{comG} - cfp(Neo^R/Kan^R)$ |
| $gltA :: P_{rpsD} - comS, P_{comG} - comS(Plm^R)$ |

# 6 Cell Differentiation Behavior

*Bacillus subtilis* exhibits two main stress responses: 1. competence and 2. sporulation. Competence is a transient differentiation program allowing for exogenous DNA uptake and integration, whereas sporulation is a terminal differentiation state where the cell can remain dormant. The interaction between sporulation and competence was previously studied [5] where it was found that the two differentiation programs independently race each other rather than using intricate cross-regulation between the two differentiation circuits. It was also discovered that cells could undergo both competence and sporulation programs simultaneously to create dual-activity cells. It was found that sporulation could exclude competence via physical mechanisms, e.g. lysing the mother cell and thus preventing competence. Furthermore, it was also found that competence could exclude sporulation in that $ComK$ expressed from sporulation promoters prevented the progression of sporulation.

The implication for this study is that by increasing the competence frequency we simultaneously suppress the sporulation rate. Spores are seen in both the Native and SynEx strains. For the purpose of the analysis of competence, spores are excluded from the analysis though the mother cell of the spore is still analyzed until lysis occurs since competence continues to be possible until lysis.

Furthermore, cells that remain in competence for an extended duration cannot divide due to suppression of *ftsZ*. This results in long, worm-like cells as the cells continue to grow in biomass without dividing. Because of this the comparison between a stable competence states and transient competence states is difficult to do on a single cell basis. To compare the different levels of induction, we rather identify the location of cells (not spores) using phase contrast microscopy, create a mask based on edge detection and the region properties of the edge enclosed regions, and then use that mask to measure the distribution of fluorescence for the area where the cells were identified. Several images and their associated masks are included as examples.

# 7 Image Analysis

Image analysis is done in the following steps:

1. Edge detection is done using the MATLAB function, *edge*, using the Laplacian of Gaussian kernel to discover zero crossings. A tolerance threshold of zero is specified to obtain all possible edges resulting in over segmentation.
2. Edges are dilated to create enclosed areas.
3. The properties of those regions are computed based on the phase contrast image, for properties such as MinIntensity, MeanIntensity, MaxIntensity, and Solidity.
4. The range of these properties is manually selected to select for the dark phase contrast areas that correspond to areas where *Bacillus subtilis* exists, excluding the phase bright spores.

5. The selected areas are then combined into a binary mask of cell locations, and the fluorescence of cfp as a reporter for $P_{comG}$ activity, which in turn is an effective reporter for $ComK$ activity, is obtained.

6. The fluorescence within the mask is then plotted as a histogram for comparison with IPTG conditions.
