# Supplementary figures and images for: Noise Expands the Response Range of the Bacillus subtilis Competence Circuit
Source: PLoS Comput Biol. 2016 Mar 22;12(3):e1004793. doi: 10.1371/journal.pcbi.1004793 (PMC4803322; doi:10.1371/journal.pcbi.1004793)

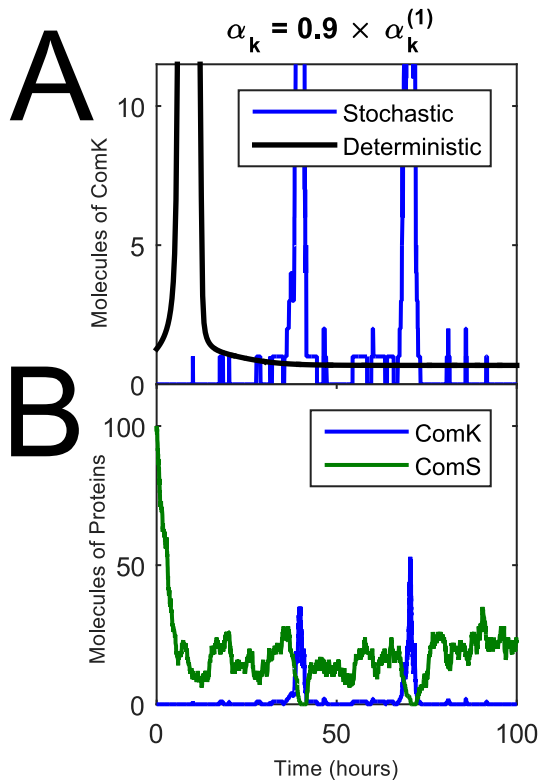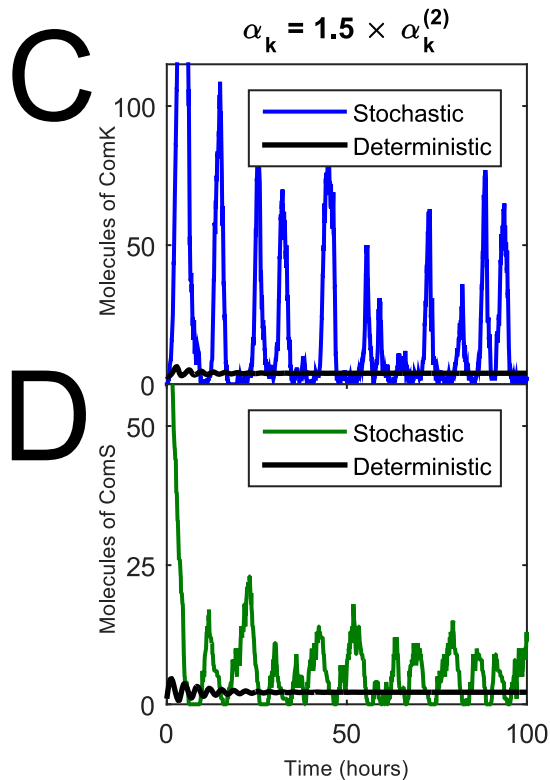

Supplement: S1 Fig — (A) Zoom-in of the top left panel in Fig 4A, showing fluctuations at low molecule number. (B) Overlay of the ComK and ComS dynamics in the left column of Fig 4A, demonstrating that ComK and ComS excitations are synchronized, with maxima in ComK slightly preceding minima in ComS. (C, D) As in the left column of Fig 4B, except less far outside the deterministically oscillatory regime (1.5 times the deterministic transition value of αk, instead of 15 times). In this regime, the deterministic dynamics are clearly damped oscillatory (black), while the stochastic dynamics are, as in Fig 4B, pseudo-oscillatory and not damped. (PDF) [file pcbi.1004793.s002.pdf]

**A**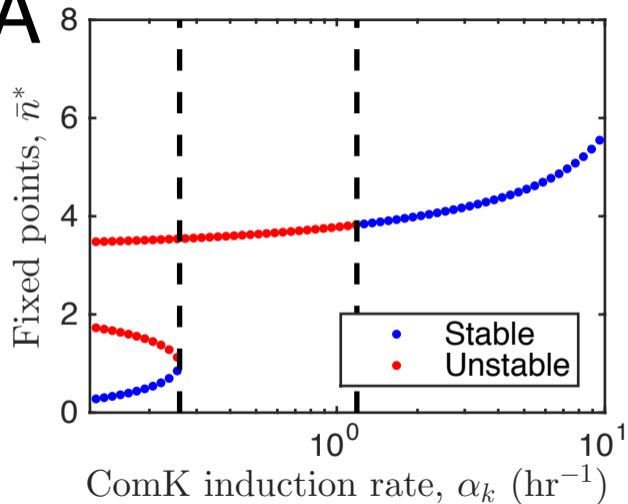**B**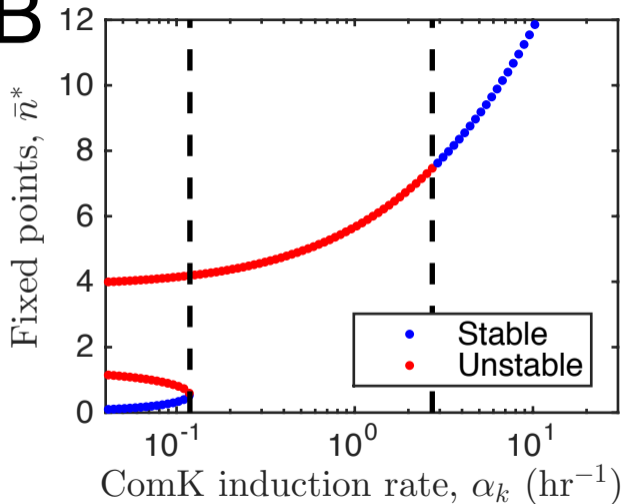

Supplement: S2 Fig — Fixed points n¯* satisfying the steady state of Eqs 6 and 7 for (A) the native circuit and (B) the SynEx circuit. Each fixed point is stable if the real parts of the eigenvalues of the Jacobian matrix evaluated at that point are negative, and unstable otherwise. The Jacobian matrix is Jij = ∂Fi/∂xj, where x1≡n¯, x2≡m¯, F1≡dn¯/dt, and F2≡dm¯/dt. For both circuits, there are three dynamic regimes. The excitable regime (low αk) is has three fixed points, one of which is stable. The oscillatory regime (intermediate αk) has one unstable fixed point. The mono-stable regime (high αk) has one stable fixed point. In the mono-stable regime, near the oscillatory regime, the eigenvalues are complex, indicating damped oscillations (see also S1 Text section 2). (PDF) [file pcbi.1004793.s003.pdf]

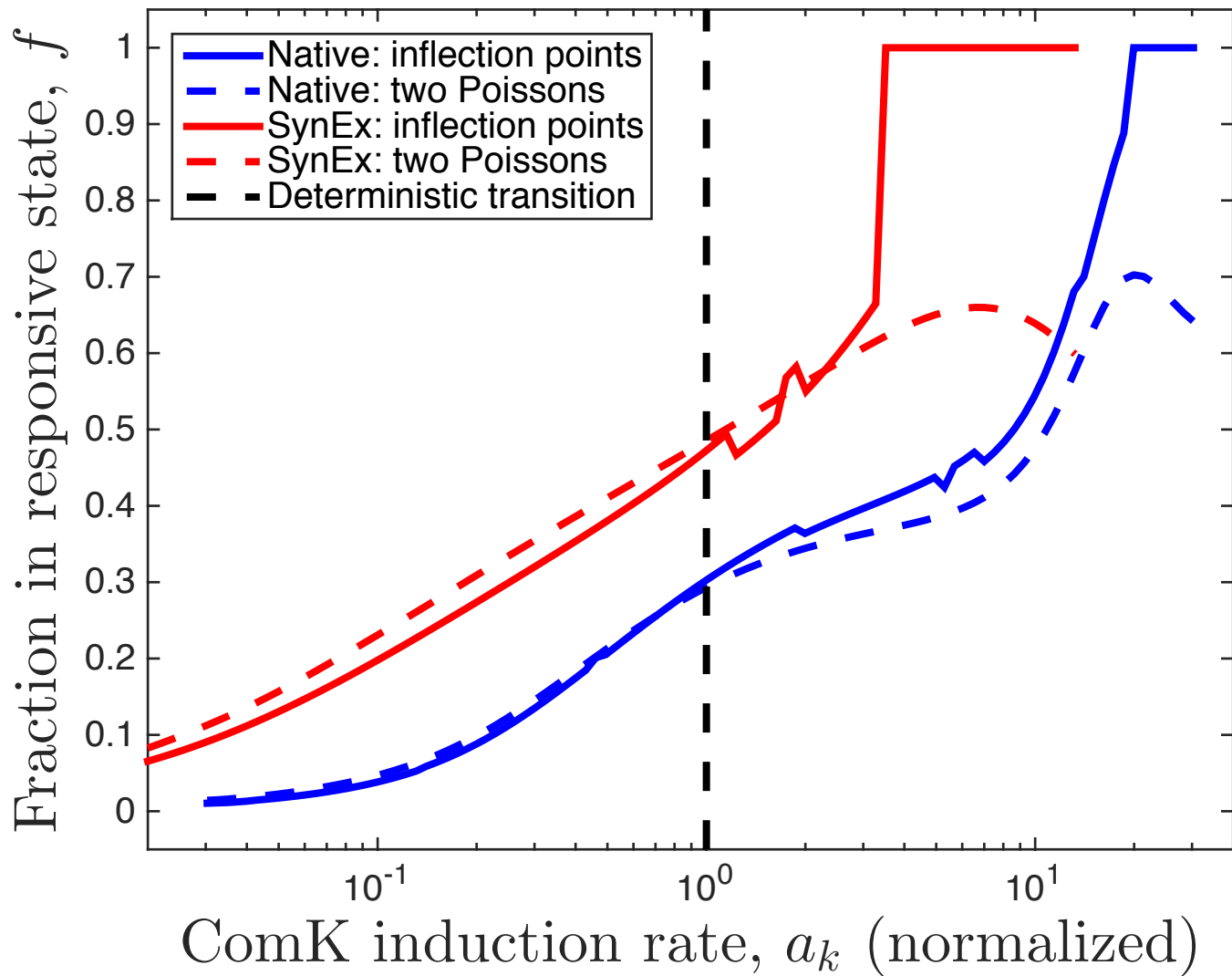

Supplement: S4 Fig — Two independent methods are used to determine f: finding the inflection points, and fitting to a mixture of two Poisson distributions (see Materials and Methods). For both the native and SynEx circuit, we see that the two methods give results that correspond very closely to each other. The two-Poisson method does not capture the transition to f = 1, since a roughly equal mixture of two Poisson distributions with similar means (f ∼ 0.5) will always provide a better fit than a single Poisson distribution (f = 1). (PDF) [file pcbi.1004793.s005.pdf]

# Native

# SynEx

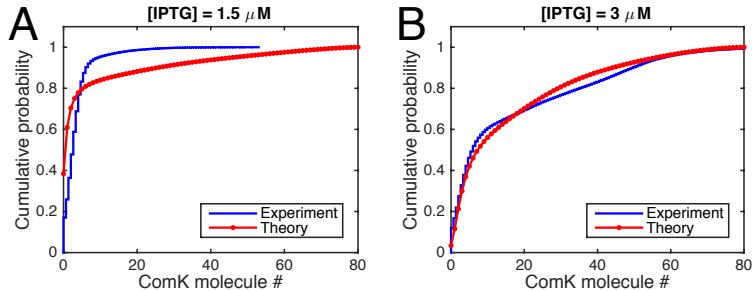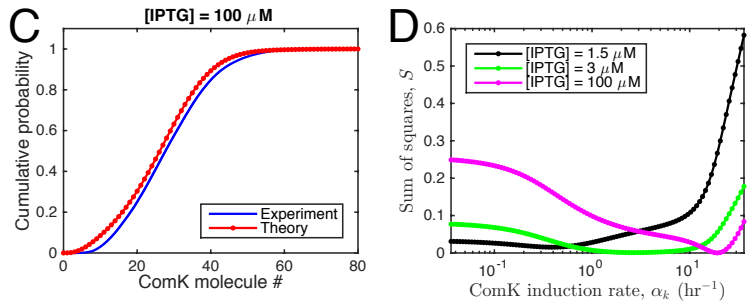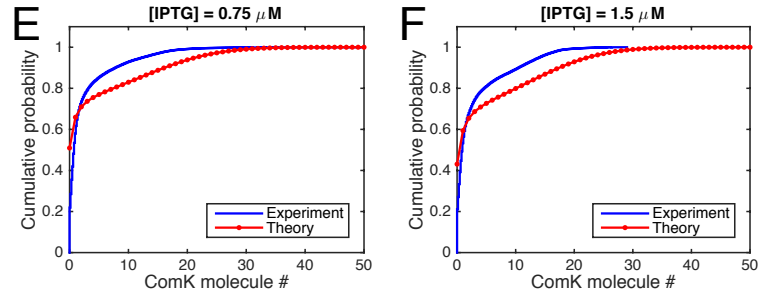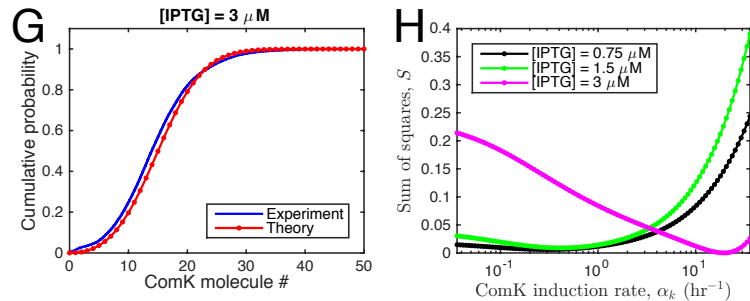

Supplement: S5 Fig — Cumulative probability distributions of fluorescence data are fit to the distributions from the stochastic model (Eqs 1–5) by minimizing the sum of squared errors, for the (A-D) native and (E-H) SynEx circuits, as described in Materials and Methods. (PDF) [file pcbi.1004793.s006.pdf]

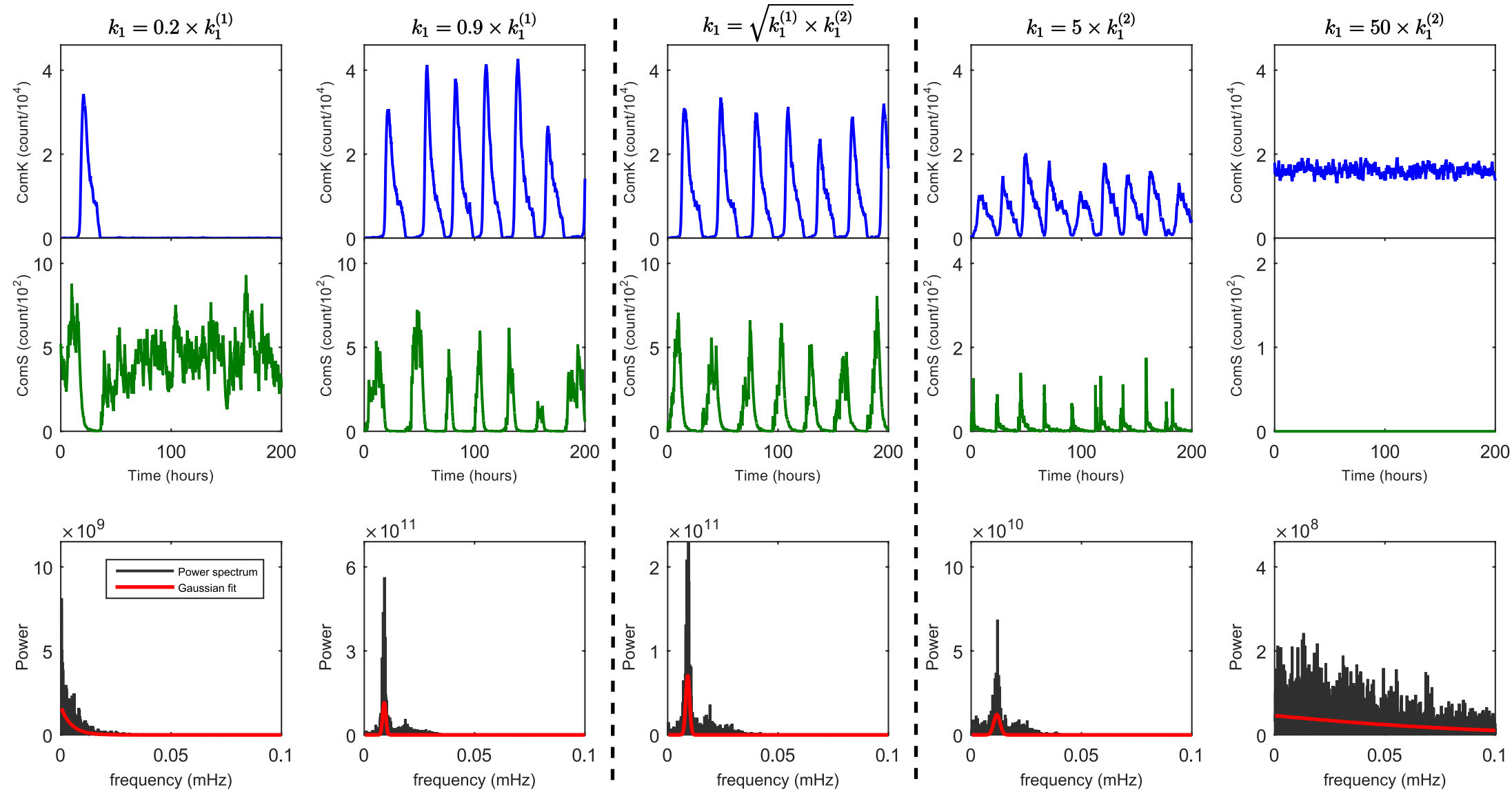

Supplement: S6 Fig — Top two rows show ComK and ComS time series from Gillespie simulations of the relaxed model that includes mRNA and competitive degradation dynamics (see S1 Text, section 3, native circuit). Far from the deterministic boundaries of the control parameter, k1(1) and k1(2) (indicated by the dashed vertical lines), the dynamics are excitable, oscillatory, and mono-stable as predicted (columns 1, 3, and 5, respectively). However, near the boundaries, but outside the oscillatory regime, noise causes oscillations to persist, due to either repeated excitations (column 2) or prevention of damping (column 4), confirming the effects seen in the reduced model of the main text. The persistence of oscillations is verified by computing the power spectrum P(ω)=|n˜(ω)|2 from the Fourier transform of the ComK time series n(t). For periodic signals, the power spectrum is peaked at a non-zero frequency ω (and in some cases its harmonics). Red line is a Gaussian fit to aid the eye. (PDF) [file pcbi.1004793.s007.pdf]

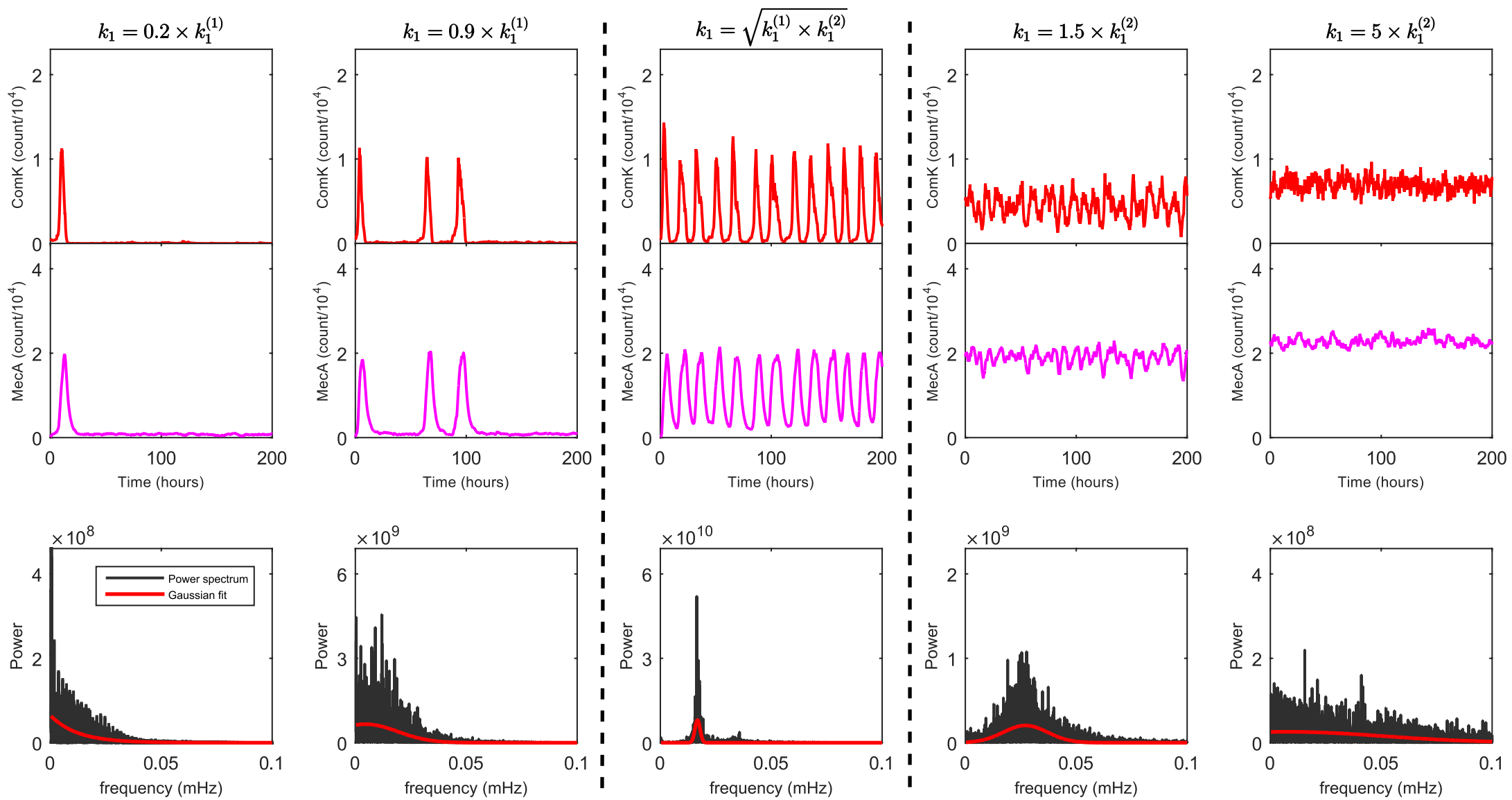

Supplement: S7 Fig — As in S6 Fig but for the SynEx circuit (see S1 Text, section 3, SynEx circuit). Once again, oscillations persist outside the deterministic boundaries as indicated by the peaked power spectra. Note, however, that oscillations are damped at the value k1=5k1(2) here, whereas in the native circuit they persist beyond this value (see S6 Fig). This confirms the effect seen in the main text that the the prevention of damping is more pronounced in the native circuit than in the SynEx circuit. (PDF) [file pcbi.1004793.s008.pdf]

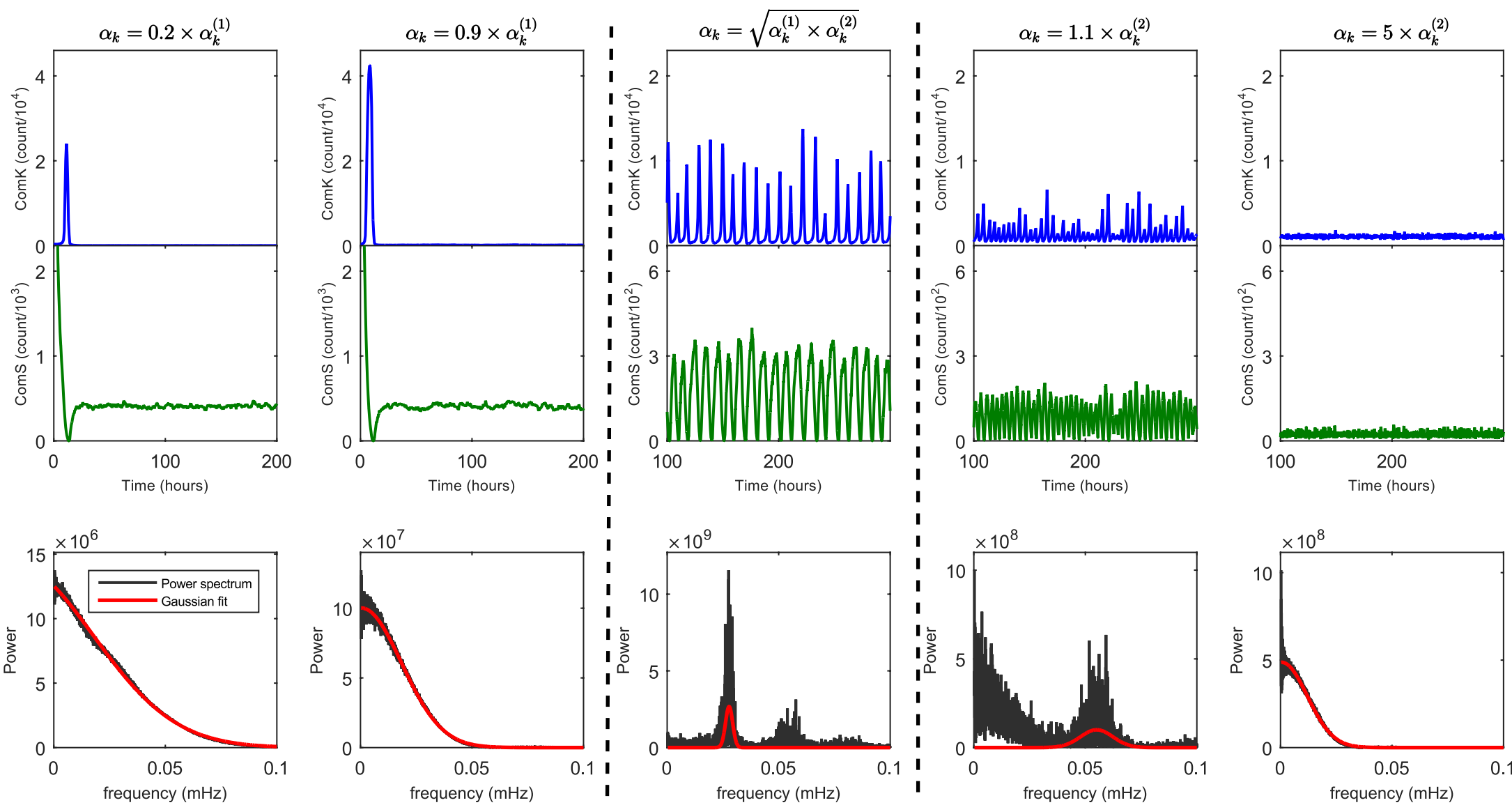

Supplement: S8 Fig — Gillespie simulations of the adiabatically reduced model of the native circuit (Eqs 1–5, as in Fig 4), but for high molecule numbers (Γk = 25000 and Γs = 20). We see that 10% outside the deterministically oscillatory regime, the stochastic dynamics are either non-oscillatory (column 2) or weakly oscillatory (column 4). This is in contrast to the low-molecule-number regime (Fig 4), where oscillations persist in these regions and beyond. We conclude that raising molecule number in the adiabatic model of the native circuit reduces the stochastic behavior to the deterministic behavior. (PDF) [file pcbi.1004793.s009.pdf]

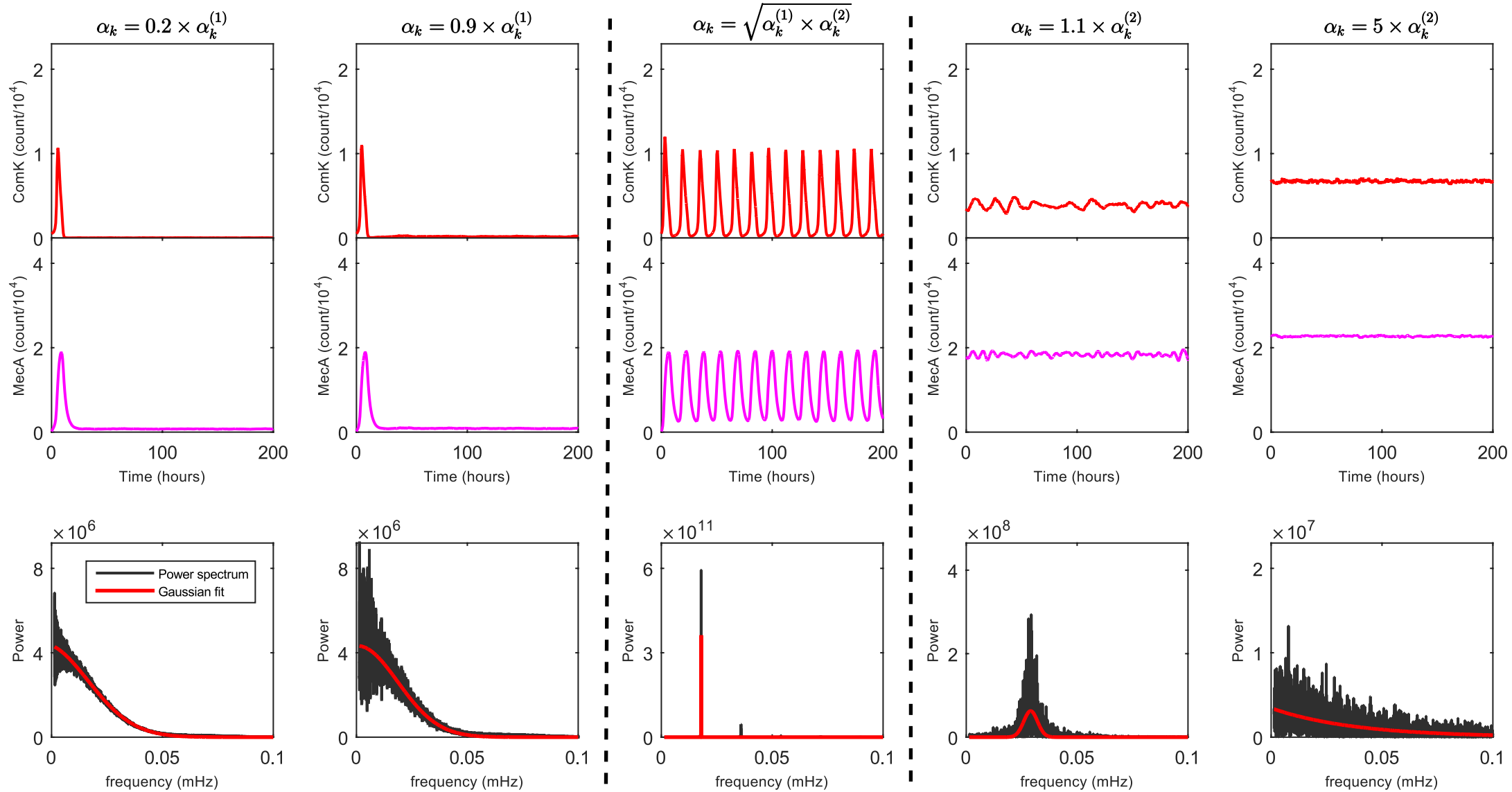

Supplement: S9 Fig — As in S8 Fig, but for the adiabatically reduced model of the SynEx circuit (Eqs 1–5) at high molecule numbers (kk = 5000 and km = 2500). Comparing to Fig 4, we similarly conclude that raising molecule number in the adiabatic model of the SynEx circuit reduces the stochastic behavior to the deterministic behavior. (PDF) [file pcbi.1004793.s010.pdf]

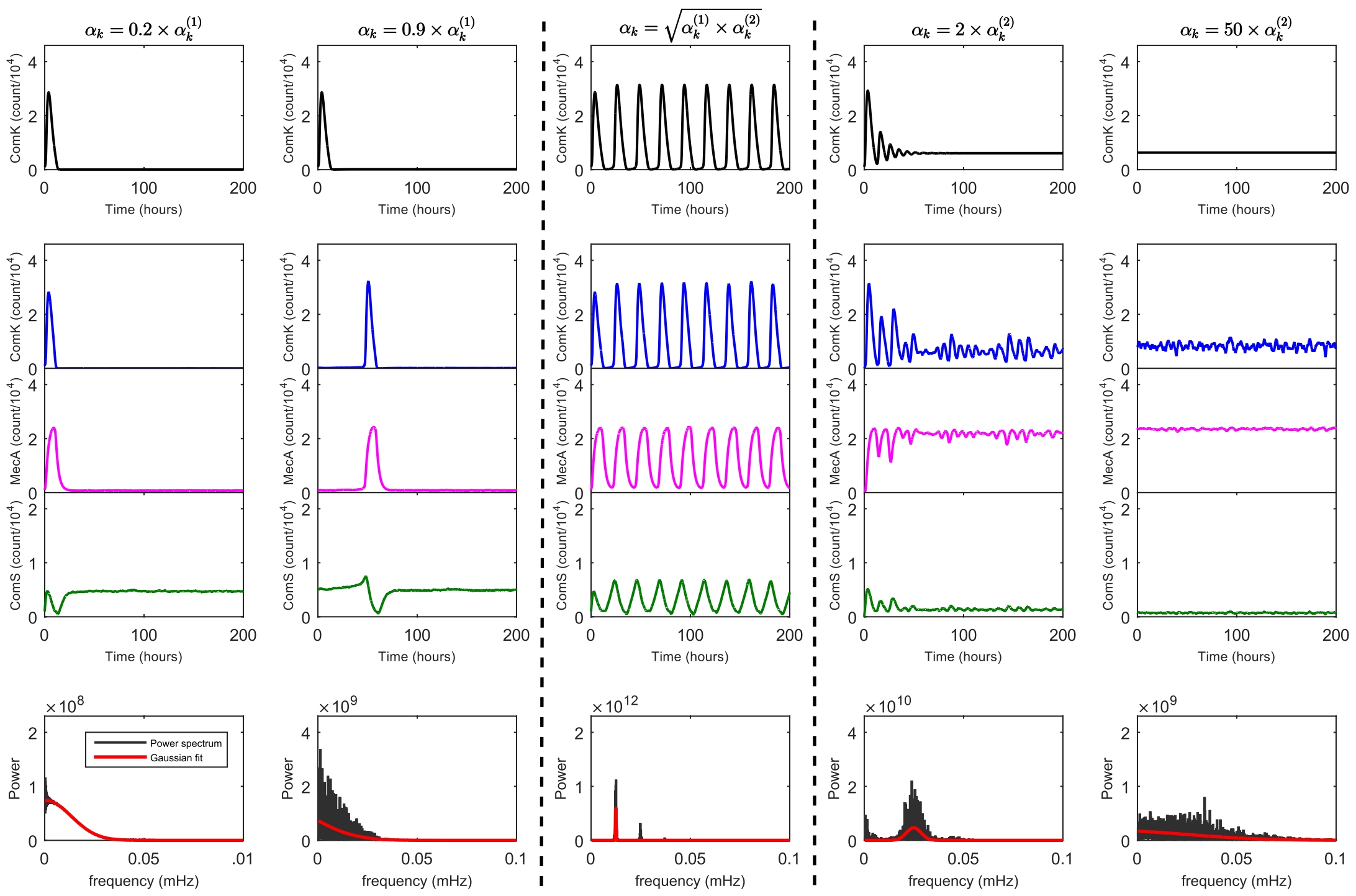

Supplement: S10 Fig — Top row shows the deterministic ComK time series from the SynExSlow model (see S1 Text, section 3, SynExSlow circuit), while the next three rows show the stochastic ComK, MecA, and ComS time series for the same model. Although the SynExSlow model only exhibits a damped oscillatory regime at these parameters, not a standard oscillatory regime (see S1 Text), we define a heuristic boundary αk(2)=0.15/s below which oscillations are not appreciably damped within the first 24 hours (column 3), and above which they are (column 4). We see that, as in S7 Fig, noise prevents damping at large values of the control parameter, even at high molecule numbers (column 4). However, as in S9 Fig, noise does not induce repeated excitations at small values of the control parameter (column 2). We conclude that the former effect is more robust. (PDF) [file pcbi.1004793.s011.pdf]

Native

SynEx

Pixel counts /  $10^3$

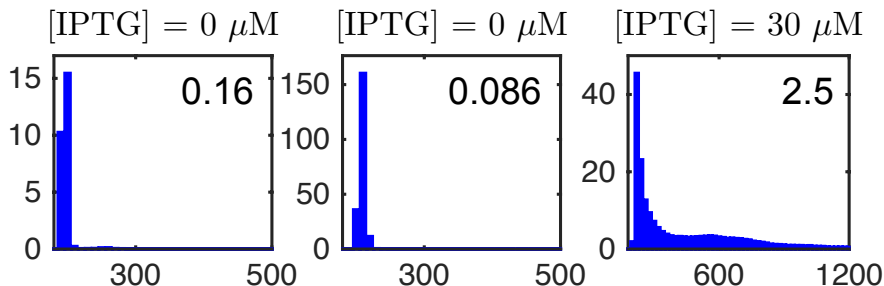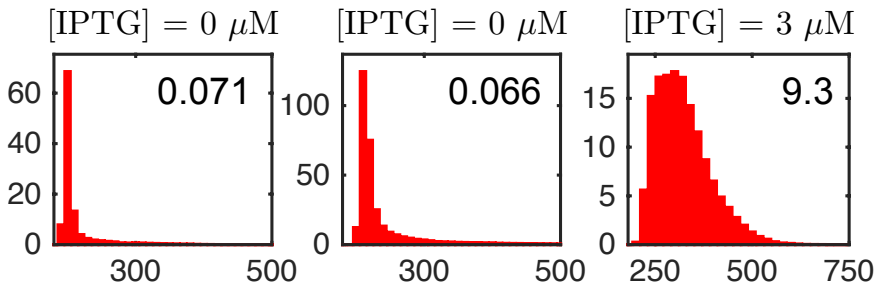

CFP fluorescence (reporter for ComK activity; AU)

Supplement: S11 Fig — Together with Fig 5B, these distributions provide the data analyzed for Fig 5C. As in Fig 5B, the normalized αk values are shown for each distribution in the upper-right corners. (PDF) [file pcbi.1004793.s012.pdf]

Stress

Stress [IPTG]

0.00  $\mu\text{M}$

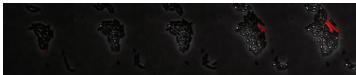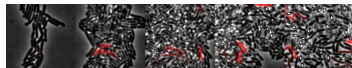

0.75  $\mu\text{M}$

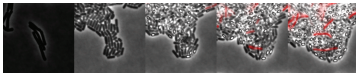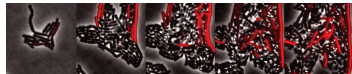

1.50  $\mu\text{M}$

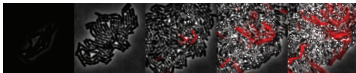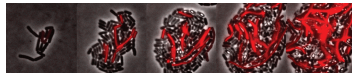

3.00  $\mu\text{M}$

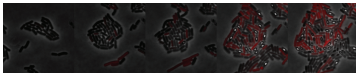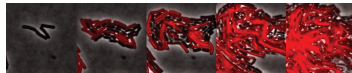

0

6

12

18

24

Hours

0

6

12

18

24

Hours

Supplement: S12 Fig — Composite phase contrast and fluorescence images of the Native and SynExSlow strains are shown with IPTG concentrations of 0, 0.75, 1.5, and 3 μM at 0, 6, 12, and 24 hours. Fluorescence represents ComK activity as reported by PcomG − cfp. (PDF) [file pcbi.1004793.s013.pdf]
