# Supplementary material for: Noise Expands the Response Range of the Bacillus subtilis Competence Circuit
Source: PLoS Comput Biol. 2016 Mar 22;12(3):e1004793. doi: 10.1371/journal.pcbi.1004793 (PMC4803322; doi:10.1371/journal.pcbi.1004793)
Supplement: S3 Fig — Fluorescence histograms are generated via isolating cells from the background by identifying connected and contiguous areas, applying a binary mask, and binning the resulting pixel intensities. Only pixels that fall within cells, not the background, are included. (PDF) [file pcbi.1004793.s004.pdf]

1. Filter connected areas based on region properties

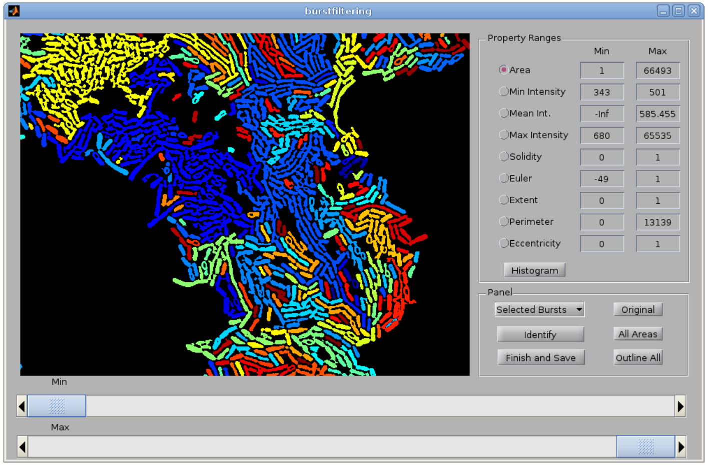

2. Contiguous areas are colored the same

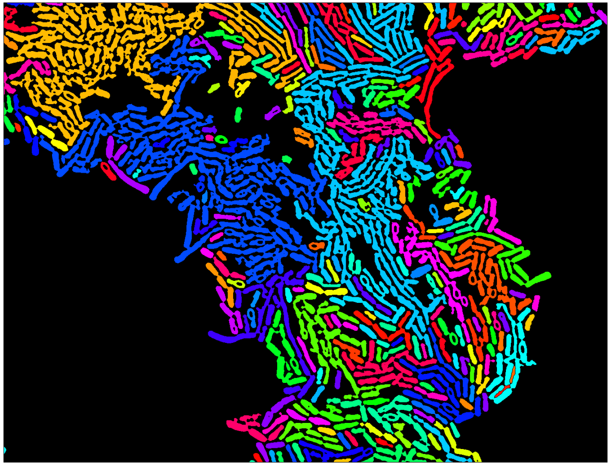

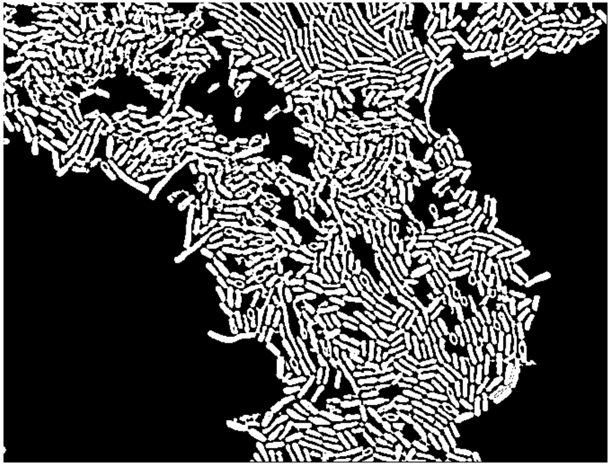

3. Remove coloring to get binary mask

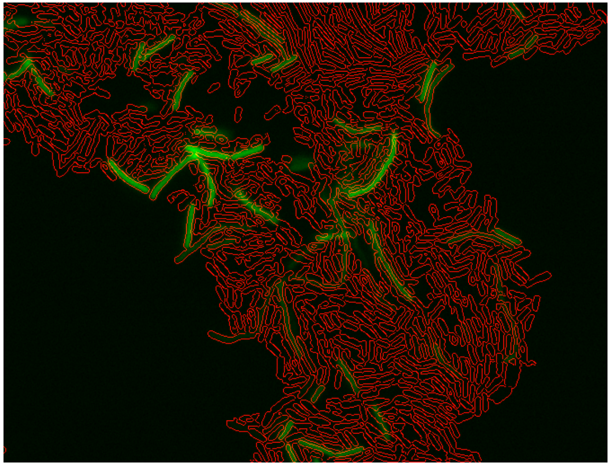

4. Apply binary mask to fluorescence image to generate pixel based histogram
